# Bronchopulmonary dysplasia: signatures of monocyte-macrophage reactivity and tolerance define novel placenta-lung endotypes

**DOI:** 10.1038/s41390-025-04025-w

**Published:** 2025-04-03

**Authors:** Karen K. Mestan, Abhineet M. Sharma, Sarah Lazar, Sonalisa Pandey, Mana M. Parast, Louise C. Laurent, Lawrence S. Prince, Debashis Sahoo

**Affiliations:** 1 Department of Pediatrics, University of California San Diego, La Jolla, CA, USA; 2 Department of Pediatrics, Divisions of Neonatology and Pediatric Pulmonology, University of Nebraska College of Medicine, Omaha, NE, USA; 3 Department of Pathology, University of California San Diego, La Jolla, CA, USA; 4 Department of Obstetrics, Gynecology and Reproductive Sciences, University of California San Diego, La Jolla, CA, USA; 5 Department of Pediatrics, Stanford University, Stanford, CA, USA; 6 Department of Computer Science and Engineering, Jacob’s School of Engineering, University of California San Diego, La Jolla, CA, USA

## Abstract

**BACKGROUND:**

Bronchopulmonary dysplasia (BPD) is a complex disease involving aberrant immune responses across the lifespan, but these mechanisms are challenging to follow in human infants. Leveraging novel Signatures of Macrophage Reactivity and Tolerance (SMaRT), we hypothesized that distinct profiles of immune cell polarization in blood and lung are associated with BPD.

**METHODS:**

Published transcriptomic datasets of cord blood-derived monocytes (CB-MNC), peripheral blood monocytes (PBMC) and tracheal aspirate-derived lung macrophages were linked to placental inflammatory (PID) and vascular (PVD) disease states using Amsterdam criteria, and BPD outcomes using NIH consensus criteria. Datasets were integrated using SMaRT to investigate monocyte-macrophage polarization tracked over the neonatal course.

**RESULTS:**

At birth and day 1 (D1), CB-MNCs and lung macrophages exhibited significant reactivity with PID versus PVD. After D14, macrophages from PID versus PVD-exposed infants exhibited reactive phenotypes (*p* = 0.002), with convergence towards original placental disease. Macrophages exhibited reactivity with BPD on D1-D7 (*p* = 0.007), but no difference after D14. At birth, CB-MNCs from BPD patients exhibited tolerance, which persisted in PBMCs throughout the neonatal period.

**CONCLUSION:**

Inflammatory versus vascular-mediated processes in developing lungs are influenced by immune cells programmed by distinct placental disease states. Circulating monocytes may play a role in attenuating macrophage reactivity towards a tolerant phenotype.

## INTRODUCTION

The placenta plays a central role in supporting fetal development, orchestrating intricate processes crucial for a successful pregnancy. Macrophages, key components of the immune system, are integral players in maintaining the delicate balance required for proper placental function. Fetal monocytes, the circulating immune cell progenitors of macrophages and other immune cells, are dynamic mediators of immune function in the early neonatal period. Recent research has increasingly focused on understanding the nuanced phenomenon of monocyte-derived macrophage polarization in a wide range of disease states, including pregnancy complications.^1–3^ However, little is still known about macrophage polarization in the fetus and its impact on the developing neonate. Placental inflammatory conditions and vascular dysfunction pose significant threats to maternal and fetal health. Dysregulation of immune responses within the placenta can lead to adverse outcomes, including preterm birth and complications such as preeclampsia and intrauterine growth restriction.^4–6^ Unraveling the complexities of macrophage polarization in the context of placental pathology and circulating fetal monocytes at birth (in cord blood) is essential for advancing our understanding of these conditions and exploring potential avenues for therapeutic intervention.

The influence of macrophage polarization on developing organ systems, particularly the lungs, is a subject of growing interest.^7–9^ In the context of neonatal outcomes, bronchopulmonary dysplasia (BPD), a common developmental chronic lung disorder affecting premature infants, has been associated with aberrant immune responses, including lung macrophage activation and polarization. Given the obvious challenges of studying the complex placenta and lung immune mechanisms during the course of early human development, novel artificial intelligence (AI) approaches that link existent rich datasets across the early lifespan are urgently needed.

Three recent mechanistic studies on immune cell regulation in placental dysfunction and BPD provide rich and novel information on the transcriptomics of monocytes and macrophages in preterm birth. Sahoo, et al, described the gene expression profiles of lung macrophages in a cohort of preterm infants at risk for BPD.^10^ They measured changes in lung macrophage gene expression in premature infants at risk for BPD, and found higher inflammatory mediator expression with BPD, and with ex vivo response to LPS stimulation. In a subsequent study and different cohorts, Sharma and colleagues reported that transcriptomic profiles in cord blood-derived fetal monocytes (CB-MNC) and their subsets (classical, intermediate, and non-classical) collected at birth reflect distinct placental dysfunction patterns from which the monocytes were drawn.^11^ Lastly, Windhorst, et al. conducted a similar study of CB-MNC subsets focusing on BPD outcomes.^12^ Collectively, the combined clinical, placental, and immune cell data from these three pivotal studies can provide insight into the mechanisms by which placental immune cell function programs early lung development and later BPD outcomes.

Taken together, the 3 cohorts and supportive data surrounding them suggest that CB-MNCs are influenced by placental dysfunction and that these altered monocyte progenitors—which in turn can influence the fate and function of early lung macrophages—are responsible for the placenta-lung crosstalk of BPD.^13^ An important gap remains whether the molecular profiles of neonatal lung macrophages themselves correlate with prior placental dysfunction. Using novel AI approaches which can predict the function of tissue-resident macrophages in any organ system, including the lung, we sought to understand how placental dysfunction might influence lung macrophage reactivity versus tolerance, and how these polarization patterns correlate or vary with human infant BPD. We hypothesized that the model would predict distinct patterns of CB-MNCs and infant lung macrophages that account for the BPD phenotype.

## STUDY DESIGN AND METHODS

### Description of datasets

Three recently published datasets were included in the analysis. These 3 studies were conducted at 3 different sites. The published transcriptomic data on tracheal aspirate-derived lung macrophages (GSE149490) and CB-MNCs (GSE195727) were used to study the relationship between lung macrophages and fetoplacental monocytes in SMaRT analysis as described below.

1. The CB-MNC dataset reported by Sharma, et al.^11^ This dataset consisted of bulk RNAseq data on monocyte subsets (classical, intermediate, and non-classical) isolated from cord blood of preterm (*N* = 59) and full-term (*N* = 11) births at the time of delivery. The main objective was to compare monocyte subset gene expression according to preterm birth and placental inflammatory and vascular dysfunction (defined below).
2. The lung macrophage dataset reported by Sahoo, et al.^10^ This study included a total of 112 preterm infants born at <30 weeks gestation. All patients were intubated for mechanical ventilation due to respiratory distress syndrome. The initial sample for each patient was obtained within the first 24 hours of life and subsequent samples were obtained weekly beginning on day 7 and continuing weekly if the patient remained intubated. Additional clinical data and placental pathology were linked to the datasets via medical chart reviews conducted by the research team at UCSD (SL and KM).
3. The CB-MNC dataset published by Windhorst, et al.^12^ This study enrolled 30 preterm infants born at <32 weeks gestational age with the collection of cord blood for monocyte isolation into subsets (classical, intermediate, non-classical) as similarly isolated and sequenced by Sharma, et al.

### Determination of BPD

In all 3 studies, the NIH consensus definitions using endpoints of oxygen requirement at 36 weeks postmenstrual age were used to identify infants with BPD.^14^

### Categories of placental dysfunction

**Fig. 1:**
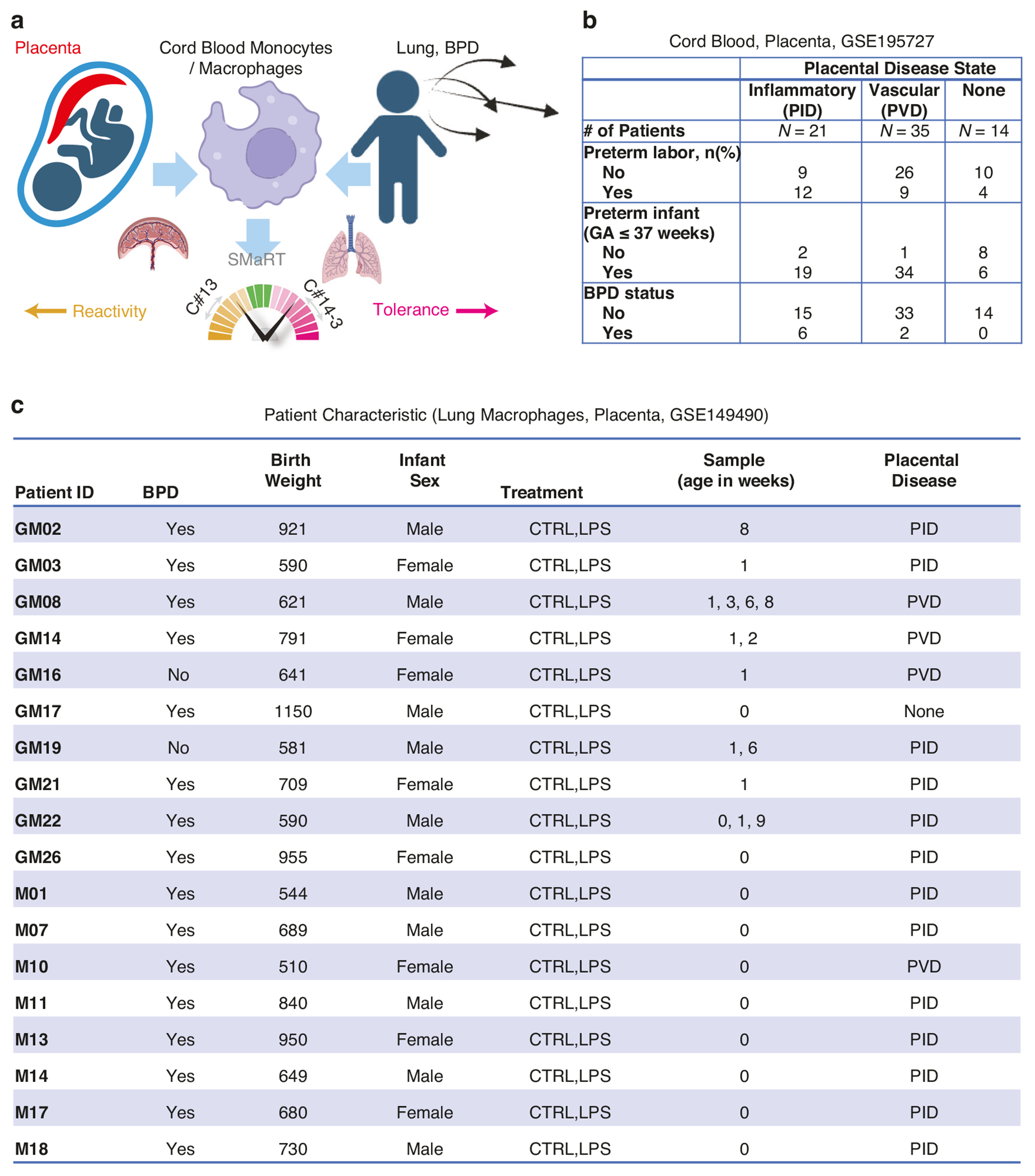
Study Design and Patient Characteristics. **a** Schematic experimental design that involves placenta phenotypes, CB-MNCs, lung macrophages and BPD. **b** Patient characteristics of the cord blood study GSE195727. **c** Patient characteristics of the lung macrophage study GSE149490 with placenta phenotypes. CTRL=control; LPS=lipopolysaccharide; PID=placental inflammatory disease; PVD=placental vascular disease. Timepoint of sampling is age in weeks: 0 = < 24 hours of life; 1 = at 1 week of life, 2 = at 2 weeks of life, up to 9 = at 9 weeks of postnatal life.

All patients in the monocyte study by Sharma, et al. had complete placental pathology data collected as part of the original study design. A comprehensive database of acute inflammatory, chronic inflammatory, maternal vascular, and fetal vascular lesions was extracted from standardized pathology reports available via electronic medical records. The details of this approach have been previously published, and are based upon the Amsterdam Placental Workshop criteria.^11,15–17^ There were no placental data collected in the original lung macrophage study by Sahoo, et al.; however, placental pathology data are available from the electronic medical records for infants born at UCSD. Thus, under IRB-approved protocols, we obtained pathology reports for the infants born at UCSD and linked the placental data to the lung macrophage database. We applied the approach used in the CB-MNC study by Sharma, et al. to assign primary placental domains for each of the 18 patients in the lung macrophage database (Fig. 1c).

Each placenta was classified into 1 of 5 primary placental domains (acute inflammation, chronic inflammation, maternal vascular malperfusion, fetal vascular malperfusion, and none), based on the presence and severity of gross and histologic lesions identified by the standard placental pathology exam and using the Amsterdam Workshop criteria.^17,18^ Two broader categories of placental disease were used in the predictive model: Placental inflammatory disease (PID) was defined as having either acute or chronic inflammation as the primary placental domain. Placental vascular disease (PVD) was defined as having either maternal or fetal vascular malperfusion as the primary placental domain.

#### Signatures of Macrophage Reactivity and Tolerance (SMaRT).

The Signatures of Macrophage Reactivity and Tolerance (SMaRT) algorithm offers a comprehensive quantitative and qualitative framework for evaluating macrophage polarization across various tissues and conditions.^19^ The study by Ghosh, et al. unveils a gene signature remarkably conserved across diverse tissues and conditions, comprising a set of 338 genes derived from a Boolean Implication Network model of macrophages.^19,20^ This model effectively identifies macrophage polarization states at the single-cell level, encompassing a spectrum of physiological, tissue-specific, and disease contexts. Remarkably, this signature demonstrates robust associations with outcomes in several diseases, underscoring its potential as a valuable predictive tool. Boolean implication network has been used to identify universal biomarkers of macrophages earlier.^21^

The algorithm uses three clusters C#13, C#14, C#3 from the published macrophage network and uses composite scores of C#13, C#14-3, and C#13-14-3 to identify macrophage polarization states (See function getCls13, getCls14a3, getCls13a14a3, and orderData in github codebase BoNE/SMaRT/MacUtils.py and the outputs in BoNE/SMaRT/macrophage.ipynb). BoNE (Boolean Network Explorer) provides a set of computational tools for Boolean analysis of biological datasets. To summarize, C13 highlights the reactivity of monocyte/macrophage polarization, while C14-3 highlights the tolerance of monocyte/macrophage polarization. C#13-14-3 takes both reactivity and tolerance factors and combines them for an overall score to determine which process dominates in the end.

**Fig. 2:**
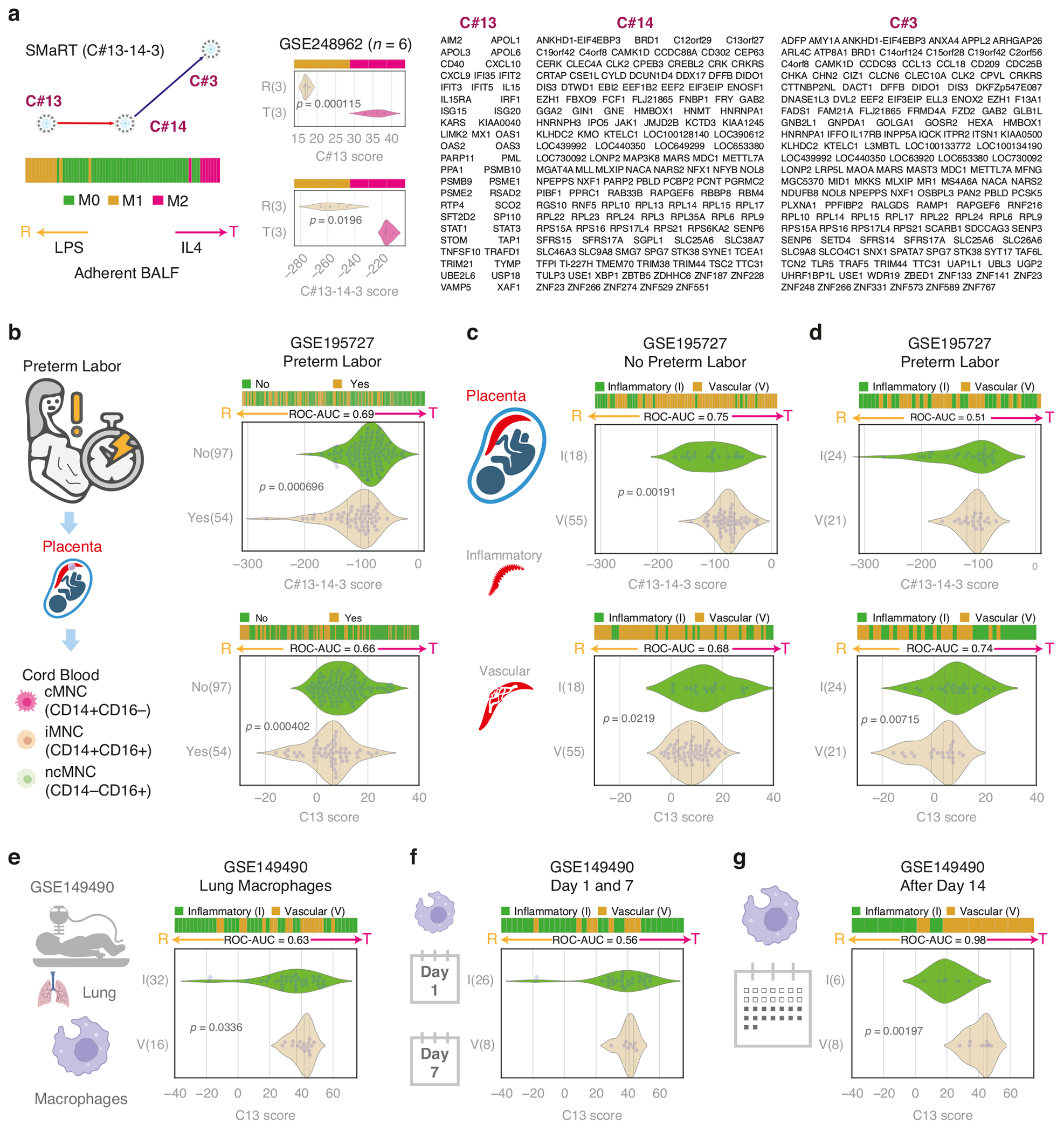
Placental vascular disease is associated with tolerant monocyte-macrophage polarization. For each figure, horizontal stacked bar plots (top) and violin (bottom) plots display the sub-classification of sample phenotypes. The x-axis shows the composite score based on clusters of genes (see Methods). Samples were ordered based on the final weighted (−1 for C#13, 1 for C#14, and 2 for C#3) and linearly combined score. Welch’s two-sample unpaired two-sided *t*-test is performed on the composite gene signature score to compute the *p*-values. Only significant *p*-values are displayed. **a** Overview of the SMaRT model for predicting macrophage/monocyte polarization (Reactive/Tolerant) using an example dataset (GSE248962). Reactive and tolerant phenotypes are interpreted relative to each other. C13-14-3 gene clusters are listed. **b** CB-MNCs are analyzed using C13-14-3 (top) as well as C13 alone (bottom) based on their preterm labor status. **c** CB-MNCs are analyzed using C13-14-3 (top) as well as C13 alone (bottom) based on their placental vascular disease status in the absence of preterm labor. **d** CB-MNCs are analyzed using C13-14-3 (top) as well as C13 alone (bottom) based on their placental vascular disease status in the presence of preterm labor. **e** Lung macrophage of infants exposed to PVD retains tolerant phenotypes. **f** No significant difference in lung macrophages between the PID and PVD groups on Day 1 and Day 7. **g** Lung macrophages after Day 14 of infants exposed to PVD retain tolerant phenotypes relative to PID (*p* = 0.00197).

To compute the composite score as described in the BoNE package, first, the genes present in each cluster were normalized and averaged (see Fig. 2a for gene lists). Gene expression values were normalized according to a modified Z-score approach centered around the StepMiner threshold (formula = (expr – SThr – 0.5)/3*stddev). A weighted linear combination of the averages from the clusters of a Boolean path was used to create a score for each sample. The weights along the path either monotonically increased or decreased to make the sample order consistent with the logical order based on Boolean Implication relationships. The samples were ordered based on the final weighted (−1 for C#13, 1 for C#14, and 2 for C#3) and linearly combined score. Performance is measured by computing ROC-AUC. For each figure generated, horizontal stacked bar plots show the ranking order of different sample types based on the composite scores of C#13, C#14-3, or C#13-14-3. Violin plots show the distribution of scores in different groups. *P*-values are computed with Welch’s Two Sample t-test (unpaired, unequal variance (equal_var = False), and unequal sample size) parameters.

#### Normalization of gene expression based on CD16/FCGR3A expression patterns.

Since the monocytes are classified based on their CD16 expression patterns, the comparison groups are expected to have uniform CD16 expression patterns. It is important to control for this variation during the analysis of monocyte/macrophage polarization. To start the normalization process, CD16/FCGR3A expression is used to adjust the BoNE composite score as defined above. First, both the BoNE composite score and macrophage gene expression are scaled for each sample type based on their dynamic range of expression values (min-max). For example, the dataset GSE220135 contains two sample types: noBPD, and BPD. Let’s take one sample from the BPD group (x, y) where x is the macrophage gene expression value and y is the original BoNE composite score. The bounding box for the BPD group demonstrates the range of values for both the BoNE composite score (S1) and the macrophage gene expression (S2). An average of BoNE composite scores and the macrophage gene expression are computed. The distance of (x, y) from the averages (S3, S4) is used to scale both values (x–S3*(S2 + 1)/(S1 + 1), y + S4*(S1 + 1)/(S2 + 1)). This process is repeated for the noBPD group. Linear regression is used to compute the trend between the transformed BoNE score and macrophage gene expression (y = mx + c). The trend is subtracted from the transformed BoNE score to compute the final normalized BoNE score (y = mx − c). Samples are now rank ordered based on the final normalized BoNE score to visualize the effect of the normalization process. Analysis of the CD16 normalized data is shown in Supplementary Fig. S1.

#### Statistical analysis.

The SMaRT gene signature is used to classify sample categories, and the performance of the multi-class classification is measured by ROC-AUC (Receiver Operating Characteristics Area Under the Curve) values. A color-coded horizontal stacked bar plot is combined with a density or violin + swarm plot to visualize the gene signature-based classification. All statistical tests were performed using R version 3.2.3 (2015-12-10). Standard t-tests were performed using python scipy.stats.ttest_ind package (version 0.19.0) with Welch’s Two Sample t-test (unpaired, unequal variance (equal_var = False), and unequal sample size) parameters. Multiple hypothesis corrections were performed by adjusting *p* values with statsmodels.stats.multitest.multipletests (fdr_bh: Benjamini/Hochberg principles). The results were independently validated with R statistical software (R version 3.6.1; 2019-07-05).

## RESULTS

### Study design to link placental disease states and lung macrophage polarization

We identified patients (Fig. 1a) with PID and PVD in previously published studies of CB-MNC (GSE195727) and lung macrophages (GSE149490).^10,11^ Three different types of CB-MNCs were profiled: classical (cMNC), intermediate (iMNC), and non-classical (ncMNC).^11^ To study macrophage polarization we employed the SMaRT model (Fig. 1a), in which the C#13 composite scores were used to identify reactivity, C#14-3 to identify tolerance states, and C#13-14-3 to identify simple overall summary of macrophage polarization.

In the CB-MNC study (GSE195727) we identified 21 patients with PID, 35 patients with PVD, and 14 patients with none of these features (Fig. 1b). We hypothesized that the monocytes from PID are more reactive compared to PVD. However, we anticipated that labor could be a confounding factor, based upon findings by Sharma, et al.^11^ To study this further, we separately analyzed the 25 patients in this cohort presenting with spontaneous preterm labor: 12 patients had PID, 9 patients had PVD, and 4 patients had normal placental pathology. Six of 21 infants with PID (29%), versus 2 of 35 with PVD (6%) developed BPD.

To link placental disease states with lung macrophage polarization data, we identified 13 patients with PID, 4 patients with PVD, and 1 patient with none of these features in the study by Sahoo, et al. (GSE149490, Fig. 1c). Lung macrophage samples were taken from intubated patients within the first 24 hours of life (week 0) and subsequent samples were obtained weekly beginning on day 7 (1 week of life) and continuing weekly (maximum 9 weeks) if the patient remained intubated.^10^ The lung macrophage samples were divided into control and LPS-treated conditions. Sixteen out of 18 patients had BPD.

### Preterm labor is associated with reactive monocyte polarization

Utilizing the SMaRT model based on C#13-14-3, CB-MNCs underwent analysis to distinguish between reactive and tolerant states (Fig. 2a). An example is provided using LPS treatment (reactive programming) and IL-4 (tolerant programming) on adherent BALF cells (GSE248962, *n* = 6). Both the C#13 score and the C#13-14-3 score significantly distinguish between the reactive and tolerant states, with the C#13 score showing greater strength than the C#13-14-3 score (Fig. 2a). A cohort of spontaneous preterm labor samples (*n* = 54) exhibited a significant association with the reactive state (*p* = 0.000696) (Fig. 2b). Notably, this correlation was evident across all monocyte populations, including classical, intermediate, and non-classical subsets. This observation aligns with the dynamic alterations in the maternal-fetal environment characterized by inflammation and tissue remodeling during preterm labor. In such an environment, monocytes are likely to undergo reprogramming, favoring adoption of a reactive state.

### Placental vascular dysfunction is associated with tolerant monocyte polarization

Applying the SMaRT model based on C#13-14-3, we investigated the association between placental disease states and the polarization state of monocytes. Notably, 18 samples from patients with PID displayed a significant reactivity score (low values) compared to the 55 samples from patients with PVD in the no preterm labor group (*p* = 0.00191) (Fig. 2c). However, no significant difference was evident in the preterm labor group, possibly attributed to macrophage influences during labor (Fig. 2d). The observed higher prevalence of reactive macrophages in patients with PID compared to those with PVD, particularly in the absence of preterm labor, underscores the dynamic nature of macrophage responses in these contexts.

### Macrophage polarization in the lung is correlated with the placental disease state

We next examined lung macrophage samples of GSE149490 through the C#13 SMART model. Control and LPS-treated cell data were combined, as there were no differences in the two treatment groups (See Supplementary Fig. S2). This is further supported by additional data showing that alveolar macrophages exhibit significantly higher reactivity compared to polarized monocyte-derived macrophages (Supplementary Fig. S3a). This suggests that lung macrophages are likely already saturated in their reactivity prior to LPS treatment. SMART analysis revealed a reactive phenotype in patients with PID (Fig. 2e, *p* = 0.0336), mirroring observations in CB-MNCs from placentas with inflammation (Fig. 2c, d). However, a more detailed analysis indicated no significant difference in samples collected on day 1 versus day 7 of life (Fig. 2f). Unexpectedly, after day 14, macrophages from PID cases exhibited a significantly reactive state compared to PVD (Fig. 2f, *p* = 0.00197). The observed persistence in polarization phenotype across longitudinally collected samples suggests a post-birth reprogramming of macrophages, that is not apparent in the first 2 weeks, but converges toward their associated placental disease phenotype after day 14. These findings highlight the dynamic nature of macrophage responses, emphasizing evidence of early immune cell programming by placental dysfunction, potential adaptation, and convergence in the postnatal period beyond the first 2 weeks of life.

### BPD is associated with reactive lung macrophages, but a tolerant blood monocyte state

**Fig. 3:**
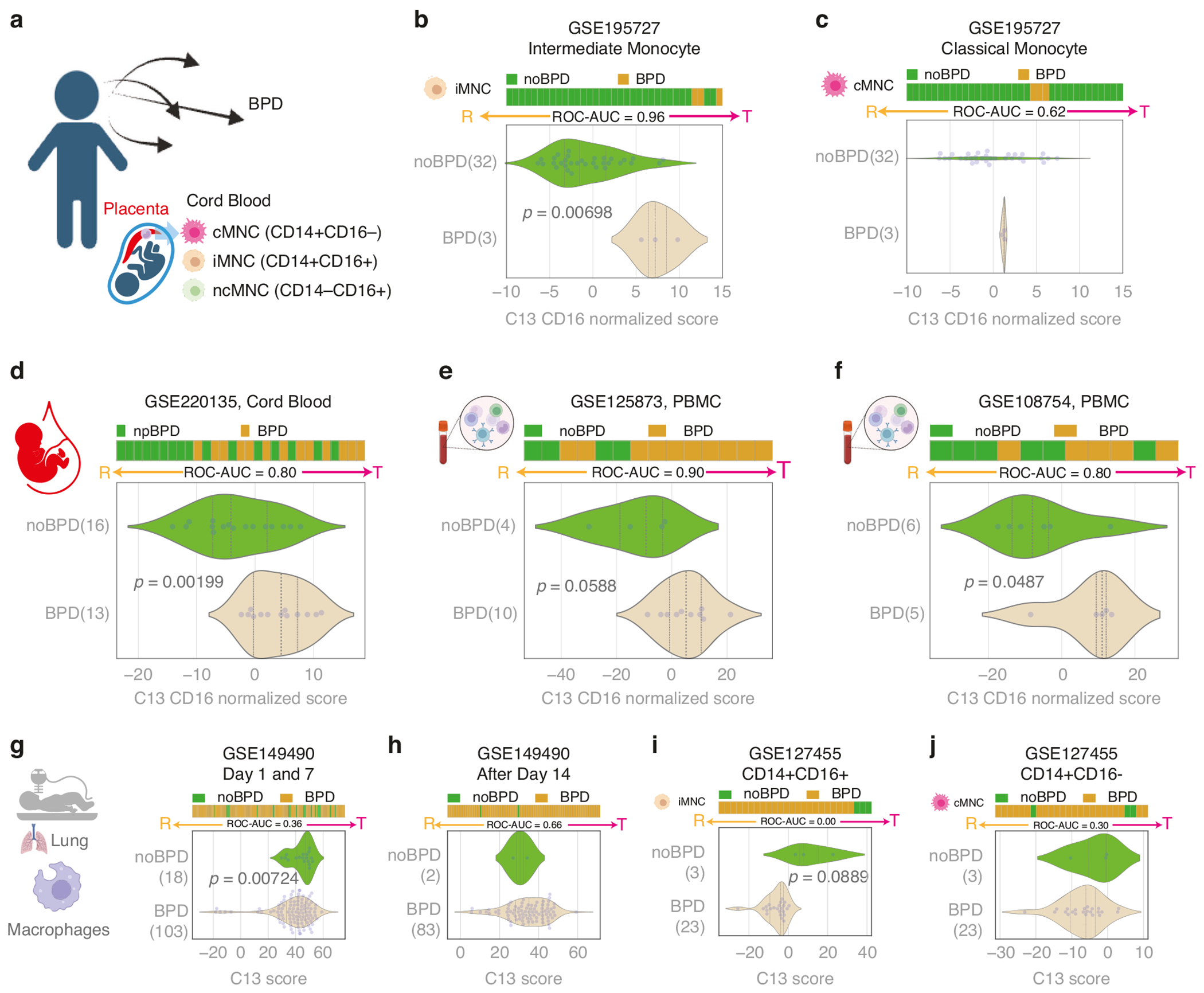
Tolerant monocytes and reactive macrophages are associated with BPD. Horizontal stacked bar plots (top) and violin (bottom) plots display the sub-classification of sample phenotypes. The x-axis shows the composite score based on clusters of genes (see Methods). Samples were ordered based on the final weighted (−1 for C#13, 1 for C#14, and 2 for C#3) and linearly combined score. Welch’s two-sample unpaired two-sided t-test is performed on the composite gene signature score to compute the *p*-values. Macrophage polarization is predicted using the C13 score. The C13 score is normalized based on CD16 (FCGR3A) expression patterns in blood monocytes and other blood datasets. **a** Study design linking CB-MNCs to BPD. Note: There were no BPD patient samples available in the dataset for non-classical monocyte analysis. **b** Tolerant polarization in intermediate CB-MNCs is associated with BPD (*p* = 0.00698). **c** Tolerant polarization in classical CB-MNCs is not significantly associated with BPD. **d** Tolerant monocyte polarization in cord blood (GSE220135) is significantly associated with BPD (*p* = 0.00199, ROC-AUC = 0.80). **e** Tolerant monocyte polarization in PBMC (GSE125873) is associated with BPD (*p* = 0.0588, ROC-AUC = 0.90) Peripheral blood was drawn at postnatal age 3 days in non-BPD controls, and at postnatal age 28 days in BPD infants.^23^ **f** Tolerant monocyte polarization in PBMC (GSE108754) is significantly associated with BPD (*p* = 0.0487, ROC-AUC = 0.80) Peripheral blood was drawn at baseline (mean postnatal age 4.6 days) for *n* = 10 infants and at BPD diagnosis (64.4 days) for *n* = 21 infants.^24^ **g** Reactive lung macrophages (Day 1 and 7 samples) are associated with BPD (GSE149490, *p* = 0.00724). **h** Lung macrophage polarization states after Day 14 are not significantly associated with BPD. **i** Sorted intermediate monocytes (CD14 + CD16+) from tracheal lavage appear more reactive in the BPD group. **j** No significant difference in classical monocytes (CD14 + CD16−) in tracheal lavage between the BPD and noBPD groups.

The cord blood study GSE195727 exhibited a relatively small number of patients with BPD (Fig. 3a). Analysis conducted using the C#13 SMaRT model revealed an association between BPD and tolerant states, specifically within the intermediate monocyte subset (Fig. 3b), contrasting with no such association observed in the classical monocyte subset (Fig. 3c). This trend persisted across three additional publicly available datasets, including CB-MNC (GSE220135)^22^ and peripheral blood monocytes (PBMC) (GSE125873, GSE108754),^23,24^ consistently demonstrating that BPD is linked to tolerant states of circulating blood monocytes (Fig. 3d–f). Collectively, these findings underscore the robust and consistent association between BPD and the induction of tolerant monocyte states. Of note, there were no BPD cases with non-classical monocyte subset data available in this cohort for comparison with the noBPD group.

The above findings were opposite of what we observed in lung macrophages of BPD patients. Using the GSE149490 dataset, examination of lung macrophage samples through the C#13 SMaRT model revealed a reactive phenotype in patients with BPD versus noBPD on days 1 and 7 (*p* = 0.00724) (Fig. 3g), but no difference from the tolerant phenotype after day 14 (Fig. 3h). To investigate whether these patterns were specific to lung macrophages versus lung monocytes, we applied the SMaRT model based on C#13-14-3 to an additional dataset of tracheal aspirate-derived isolated monocytes from BPD patients generated by Eldredge, et al. (GSE127455).^25^ A similar trend was observed in double-positive (CD14 + CD16+) lung monocytes, but no significant difference was found in either double-positive (CD14 + CD16+) or single-positive (CD14 + CD16−) lung monocytes when collected over a pooled range of 0 to 4 weeks (Fig. 3i, j). Thus, in BPD patients the reactive (pro-inflammatory) phenotype observed in lung macrophages is distinct from lung monocytes, and divergent from the tolerant phenotype of CB-MNCs at birth. As the reactive state appears most prominent at birth and in the first 2 weeks of life, we speculate that lung macrophage reactivity may be exacerbated by the transition to relative hyperoxia at birth and in the early postnatal period in patients at highest risk for BPD. The persistence of the tolerant phenotype in circulating PBMCs beyond 2 weeks suggests an active role of monocytes—in particular the intermediate subset—in attenuating the reactive state. These findings underscore the influence of monocyte heterogeneity in the pathogenesis of BPD.

### SMaRT analysis using human donor lung data of the LungMAP cohort

**Fig. 4:**
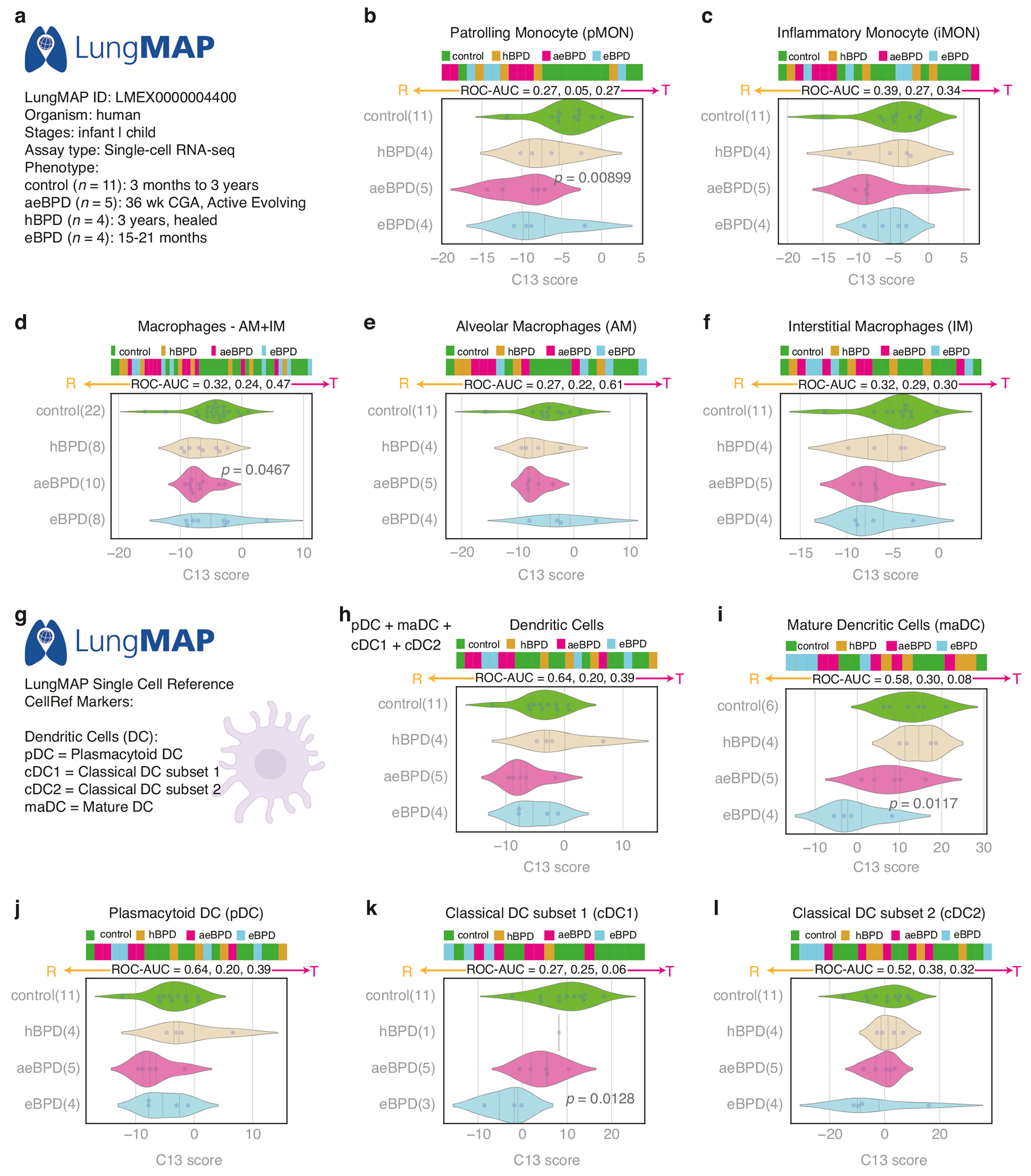
Analysis of monocytes, macrophages, and dendritic cell polarization in the LungMAP cohort. Horizontal stacked bar plots (top) and violin (bottom) plots display the sub-classification of sample phenotypes. The x-axis shows the composite C13 scores based on clusters of genes (see Methods). Welch’s two-sample unpaired two-sided t-test is performed on the composite gene signature score to compute the *p*-values. Datasets from LMEX0000004400 were accessed using the reference by Gaddis, et al.^26^ with accompanying clinical information as downloaded from LMEX0000004400 (File: Donor-Metadata.xlsx) and as described by Dylag, et al.^27^ The available datasets were generated by single-cell RNAseq performed on BPD infants (*n* = 13) and age-matched noBPD controls (*n* = 11). Healed BPD (hBPD; *N* = 4), active evolving BPD (aeBPD; *N* = 5), and established BPD (eBPD; *N* = 4) cases were compared to the control group (noBPD, healthy/normal lung histology). The single-cell RNAseq datasets were pseudobulked based on their CellRef annotations. Macrophage polarization was predicted using the C13 score. **a** Description of the study cohort from the LungMAP database. **b** Patrolling Monocytes (pMON, similar to non-classical monocytes) are reactive with aeBPD as compared with control (*p* = 0.00899). **c** Inflammatory Monocytes (iMON) are similar in function to classical monocytes. **d** Lung Macrophages, including both the alveolar and interstitial populations are reactive in aeBPD versus control (*p* = 0.0467). **e** Alveolar Macrophages. **f** Interstitial Macrophages (**g**) Different classes of the dendritic cells annotated using CellRef database. **h** Combined analysis of all dendritic cells. **i** mature dendritic cells are more reactive with eBPD (*P* = 0.0117). **j** plasmacytoid dendritic cells. **k** classical dendritic cells subset 1 were more reactive in eBPD (*p* = 0.0128). **l** classical dendritic cell subset 2.

To further investigate the above findings at a single-cell level, we leveraged publicly available datasets of the LungMAP consortium.^26^ At the time of this analysis, lung single-cell RNAseq data from 24 pediatric human donors, classified based on the history of prematurity and lung histopathology into the following phenotypic groups based upon published LungMAP consortium criteria^27^ (Fig. 4a): 5 infants with “active evolving” BPD (aeBPD) who died around 36 weeks postmenstrual age (PMA, range 46.6-85.0 weeks), 4 infants with “established” BPD (eBPD) who died between 15-21 months of age (89.9–130.3 weeks PMA), and 4 infants with “healed” BPD (hBPD) with lung autopsy at 3 years of age. For each age range, there were 11 datasets from 11 age-matched pediatric human donors without BPD (No lung disease, normal lung histology; age range: 3 months to 3 years) in which to compare aeBPD, eBPD, and hBPD with noBPD. Single cell RNAseq datasets and metadata (LMEX0000004400) were downloaded on January 24, 2025, pseudobulked based upon their CellRef annotations. Figure 4b, c shows that patrolling monocytes (pMON) but not inflammatory monocytes (iMON) were significantly reactive in aeBPD as compared with controls. Lung macrophages (alveolar and interstitial combined) were also reactive in the aeBPD group (Fig. 4d–f). We also analyzed dendritic cell subsets (Fig. 4g–i). Mature (maDC) and classical DC subset 1 (cDC1) were significantly more reactive in eBPD as compared with the control group. We provided additional data to show that dendritic cells can be polarized by HIV infection (Supplementary Fig. S3b). Collectively, these data demonstrate how SMaRT analysis can be leveraged to elucidate changes in lung immune cell types, as well as age-dependent changes in polarization during the course of evolving to resolving BPD.

## DISCUSSION

In this study, we leveraged existing databases of clinical, placental and immune transcriptomic data coupled with novel analytic tools to evaluate monocyte-macrophage polarization across cord blood and lung tissues with BPD outcomes. We found that preterm labor was associated with a reactive state of macrophages, and that this correlation is similar across monocyte subpopulations. We also found that, in the absence of preterm labor, monocytes from infants exposed to PID, characterized by histologic lesions of acute and chronic inflammation, were associated with significant reactivity scores (low values) compared to monocytes from patients with PVD (maternal and/or fetal vascular malperfusion). Examining lung macrophage samples through the C#13 SMART model, we found a reactive phenotype in patients with PID, mirroring observations in cord blood from the placenta. While these associations were not found in lung macrophages collected in the first week of life, at later time points macrophages from patients exposed to PID exhibited a more prominent reactive state compared to PVD. Collectively, these findings suggest a dichotomy between inflammatory versus vascular-mediated endotypes in the developing lung that are influenced by immune cells programmed by distinct placental disease states.

Perhaps the most intriguing findings were in the discrepancies between lung macrophage and CB-MNC polarizations in BPD versus noBPD patients. As expected, lung macrophages from BPD infants were associated with a reactive state in the early postnatal period (first 2 weeks of life). This is likely due to the heightened pro-inflammatory state of premature infant lungs upon exposure to relative hyperoxia at birth and in the first weeks of life due to high supplemental oxygen exposure, which is necessary for survival among infants who later develop BPD. We found this to be in stark contrast to the tolerant polarization state of circulating CB-MNCs drawn from the placenta at birth, which persisted in PBMCs in the postnatal period. As the polarization profiles of lung macrophages appeared to converge towards their associated placental disease state (inflammatory versus vascular) (Fig. 2f), we speculate that circulating monocytes play a role in attenuating lung macrophage reactivity towards a tolerant phenotype, particularly with placental vascular disease. If this is the case, then CB-MNC polarization states, in the context of the placental pathology from which they arise, may serve as more reliable predictors of BPD than the lung immune cellular milieu observed in the perinatal-neonatal period.

Placental inflammatory lesions, such as chorioamnionitis (acute inflammatory lesions in the chorion and amnion), and funisitis (inflammatory infiltration in the umbilical cord and thus the fetus), are the most commonly associated placental findings associated with adverse neonatal outcomes.^28–31^ The association between placental inflammation and BPD has been variably and inconsistently reported, suggesting that the link between placental disease and fetal lung programming is more complex than previously understood.^32,33^ More recent evidence has suggested that other pathophysiologic processes occurring *in utero* may lead to other forms—or endotypes—of BPD mediated by mechanisms other than intrauterine inflammation.^34–36^ For example, placental vascular dysfunction, characterized by lesions of maternal and fetal vascular malperfusion in which abnormal trophoblast implantation, failed spiral artery remodeling, decidual and/or fetal vasculopathies and other events are associated with acute or chronic fetal hypoxia. The importance of placental vascular processes on neonatal outcomes has recently been highlighted by robust associations between PVD and BPD with pulmonary hypertension.^15,37^ The findings of this study provide new mechanistic insights into how distinct forms of placental dysfunction lead to distinct endotypes of BPD.

Placental health is intricately linked to the balance of monocyte and macrophage populations, with PID often characterized by the presence of reactive macrophages. In contrast, PVD exhibits a different immune cell profile. These distinct immune cell populations circulating through the placenta have the potential to exert a lasting impact on the polarization of macrophages in other organs, particularly the lungs.^38–40^ The process of labor and birth introduces a significant shift in the immune milieu, reprogramming macrophages to accommodate the dynamic changes associated with parturition. These and other physiologic processes of preterm birth, interventions, and exposures to the NICU environment further challenge the immune system thus distorting the differences between macrophage states programmed by various placental disease conditions. Despite this, circulating monocytes that maintain a tolerant profile throughout may be responsible for the convergence of lung macrophages towards their original placental disease state in the later neonatal period.

The relationship between labor and monocyte-macrophage polarization is a complex yet understudied area of study within reproductive immunology.^41–43^ Monocytes, as key components of the innate immune system, play a crucial role in the dynamic processes associated with labor, contributing to both the initiation and resolution of this complex physiological event. During labor, the uterine environment undergoes significant changes, marked by inflammation and tissue remodeling. Macrophages within the uterine tissues exhibit a shift in their polarization states, transitioning between reactive/pro-inflammatory (M1) and tolerant/anti-inflammatory (M2) phenotypes.^44^ This dynamic polarization is orchestrated to facilitate the various stages of labor, including cervical ripening, uterine contractions, and postpartum tissue repair.

Macrophages exist in heterogeneous states within tissues, with some being reactive and others tolerant, rather than uniformly polarized. The C13 score reflects the reactive states of macrophages, while the C14-3 score represents their tolerant states. The C13-14-3 score provides a summary of the overall polarization state in a bulk setting. As suggested in Fig. 2c, an abundance of tolerant macrophages may drive the overall low reactive score. This highlights the heterogeneity of macrophages and emphasizes the need for further investigation in future studies.

By examining the immune cell landscape of placentas before labor, via the CB-MNC progenitors drawn from placental blood at C-section births, we gain insight into the initial conditions that influence the trajectory of macrophage polarization in distant organs, such as the lungs. This long-term influence on lung macrophages is particularly relevant in the context of BPD. Understanding how fetal monocytes, specifically those in an inflammatory state, may contribute to the programming of lung macrophages provides a unique perspective on the etiology of BPD. The consistent reflection of pre-labor fetal monocytes in the programming of lung macrophages suggests a potential link between placental health and the development of respiratory conditions in neonates. Further research in this direction may uncover novel therapeutic strategies aimed at modulating macrophage behavior to mitigate the risk of BPD and improve neonatal outcomes.

The more recently generated single-cell RNAseq data available through the LungMAP consortium (Fig. 4) provides additional insight into the role of macrophage reactivity and tolerance in BPD. While linkage to placental data was not feasible with these datasets, SMaRT analysis revealed important patterns of timing and transition of lung macrophages from a reactive state in active evolving BPD (around 36 weeks PMA) to a tolerant state in established and healed BPD (1-3 years of age). To some extent, these trends validate the SMaRT analysis findings observed in BAL-derived lung macrophages in which there is overall convergence to the tolerant state after D14 in vitro. An important limitation of studies using BAL-derived macrophages has been that they can only be studied from intubated infants, which precludes the ability to study these cells in different stages of BPD pathogenesis. The LungMAP datasets allowed SMaRT analysis of macrophages, as well as related immune cells such as patrolling monocytes (analogous to the non-classical subset), inflammatory monocytes (analogous to classical monocytes) as well as dendritic cells (Fig. 4). Thus, an overarching strength of this study is that it demonstrates how AI approaches such as SMaRT can be leveraged to overcome inherent limitations of multiple studies, while capitalizing on the mechanistic and histologic evidence in each, to assemble new hypotheses for future research.

In contrast to LungMAP, there were no similarly comprehensive published datasets on placental macrophage transcriptomics linked to preterm birth or BPD outcomes available at the time of this analysis. Given the highly complex and heterogeneous nature of the human placenta, gathering high-quality transcriptomic data of placental tissues for BPD research is perhaps even more challenging than for the lung. However, we anticipate that a growing number of placental and lung transcriptomic data that can be analyzed by SMaRT and other AI approaches will be available in the near future—providing even more insight into mechanisms of placenta-lung crosstalk that define BPD endotypes.

Other limitations of this study include the differences in cohort size and differences in the primary focus of each study that required retrospective collection of placental data in the in vitro lung macrophage study. Paired monocyte-macrophage data following a single cohort with complete placental pathology, cord blood, and lung specimens with BPD outcomes from the same patients would have been optimal, but such studies involving extremely premature infants have inherent challenges—in particular the collection of immune cells from lung specimens of intubated patients. Through SMaRT analysis, we were able to overcome these limitations to some extent, by linking rich transcriptomic and other datasets across different cohorts and over time. Another limitation is that the SMaRT algorithm was designed to specifically focus on macrophage polarization patterns and does not account for the myriad of other complex mechanisms involved in the pathogenesis of BPD. These include the influences of gestational age and shared genomic background effects that may be inherent to maternal disease states, such as chorioamnionitis and preeclampsia. Alternative genomic pathways associated with angiogenesis and vascular development will require other approaches, perhaps modeled after SMaRT and other AI algorithms. These novel approaches provide exciting new possibilities to elucidate mechanisms of complex diseases such as BPD that require longitudinal study of immune, vascular, and other processes across the lifespan.

In conclusion, the SMaRT analysis serves as a novel model for studying multifactorial BPD and its immune-mediated endotypes. A more comprehensive investigation of the intricate relationships between macrophage polarization and BPD mediated by placental dysfunction is needed. By shedding light on these interconnected processes, these discoveries will contribute valuable insights that inform novel therapeutic strategies for mitigating the impact of placental and lung-related pathologies on maternal and childhood health.

## Supplementary Material

supplementary info

The online version contains supplementary material available at https://doi.org/10.1038/s41390-025-04025-w.

## Data Availability

All data are available in the main text or the supplementary materials. The codes are available in https://github.com/sahoo00/BoNE.

## IMPACT:

- Bronchopulmonary dysplasia is a complex, multifactorial chronic lung disease in which the mechanisms of placenta-lung crosstalk are poorly understood.
- This study uses novel AI approaches to understand how fetal monocytes and lung macrophages contribute to the pathogenesis of BPD.
- The study identified changes in macrophage reactivity versus tolerance that could explain the heterogeneity and adaptability of immune cells and the placenta in modulating health and disease in the developing fetus and neonate.
