# Supplementary material for: Bronchopulmonary dysplasia: signatures of monocyte-macrophage reactivity and tolerance define novel placenta-lung endotypes
Source: Pediatr Res. 2025 Apr 3;98(6):2352–62. doi: 10.1038/s41390-025-04025-w (PMC12335971; doi:10.1038/s41390-025-04025-w)
Supplement: Supplementary file 1 — Supplementary Information [file 41390_2025_4025_MOESM1_ESM.pdf]

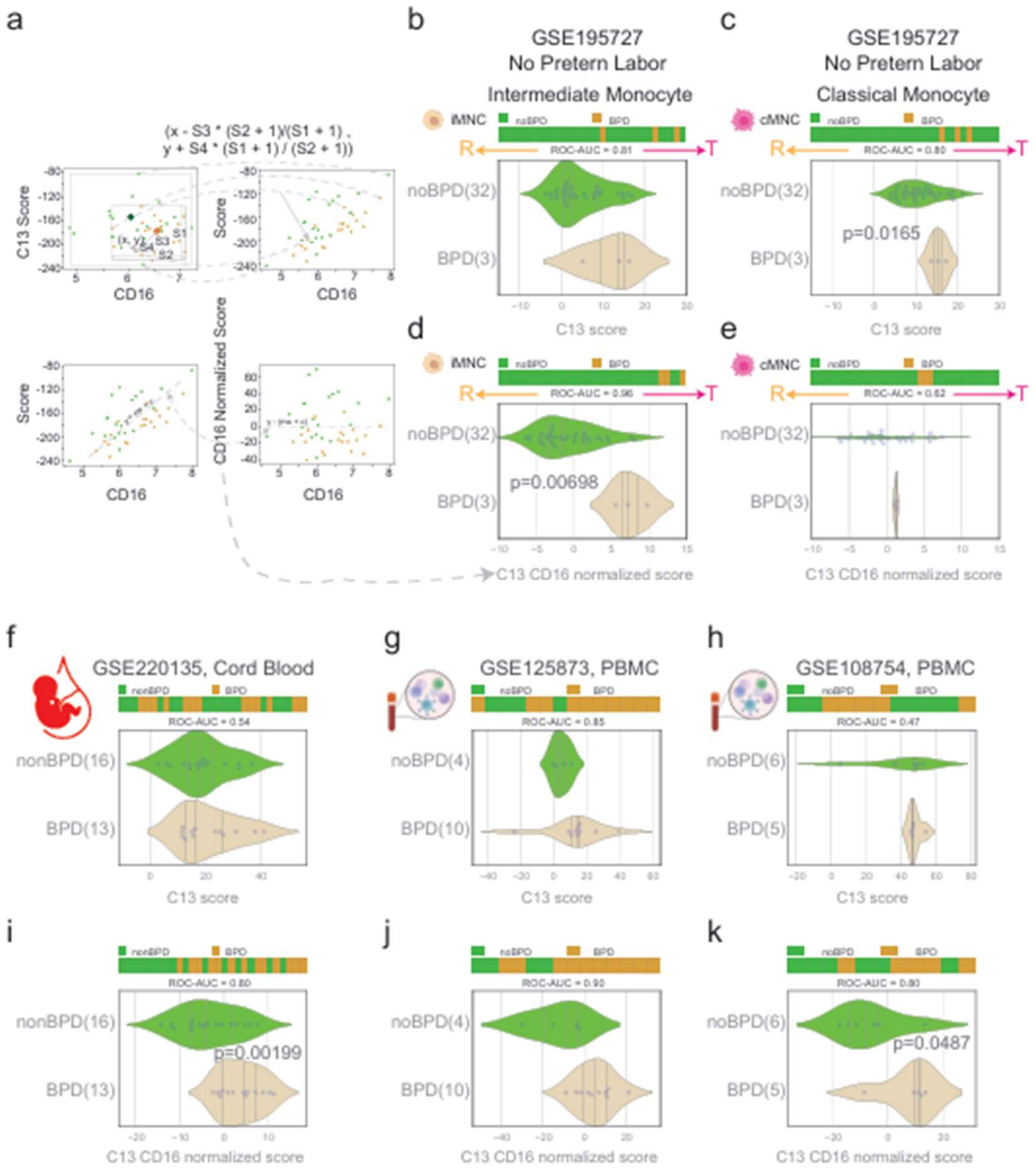

Supplemental Figure S1: Analysis of the CD16 Normalized Data. Horizontal stacked bar plots (top) and violin (bottom) plots display the sub-classification of sample phenotypes. The x-axis shows the composite score based on clusters of genes (see Methods). Welch's two sample unpaired two-sided t-test is performed on the composite gene signature score to compute the p-values. C13 score is normalized based on CD16 (FCGR3A) expression pattern in the blood monocyte and other blood datasets because the comparison groups are expected to have uniform CD16 expression patterns. Macrophage polarization is predicted using C13 score and C13 CD16 normalized score (a) Detailed approach for normalization of C13 score based on CD16 expression. (b) Analysis of intermediate cord blood monocytes with no preterm labor using C13 score. (c) Analysis of classical cord blood monocytes with no preterm labor using C13 score. (d) Analysis of intermediate cord blood monocytes with no preterm labor using C13 CD16 normalized score. (e) Analysis of classical cord blood monocytes with no preterm labor using C13 CD16 normalized score. Additional analysis of cord blood (f, i) and PBMC samples (g, h, j, k) using both C13 score (f, g, h) and C13 CD16 normalized score (I, j, k).

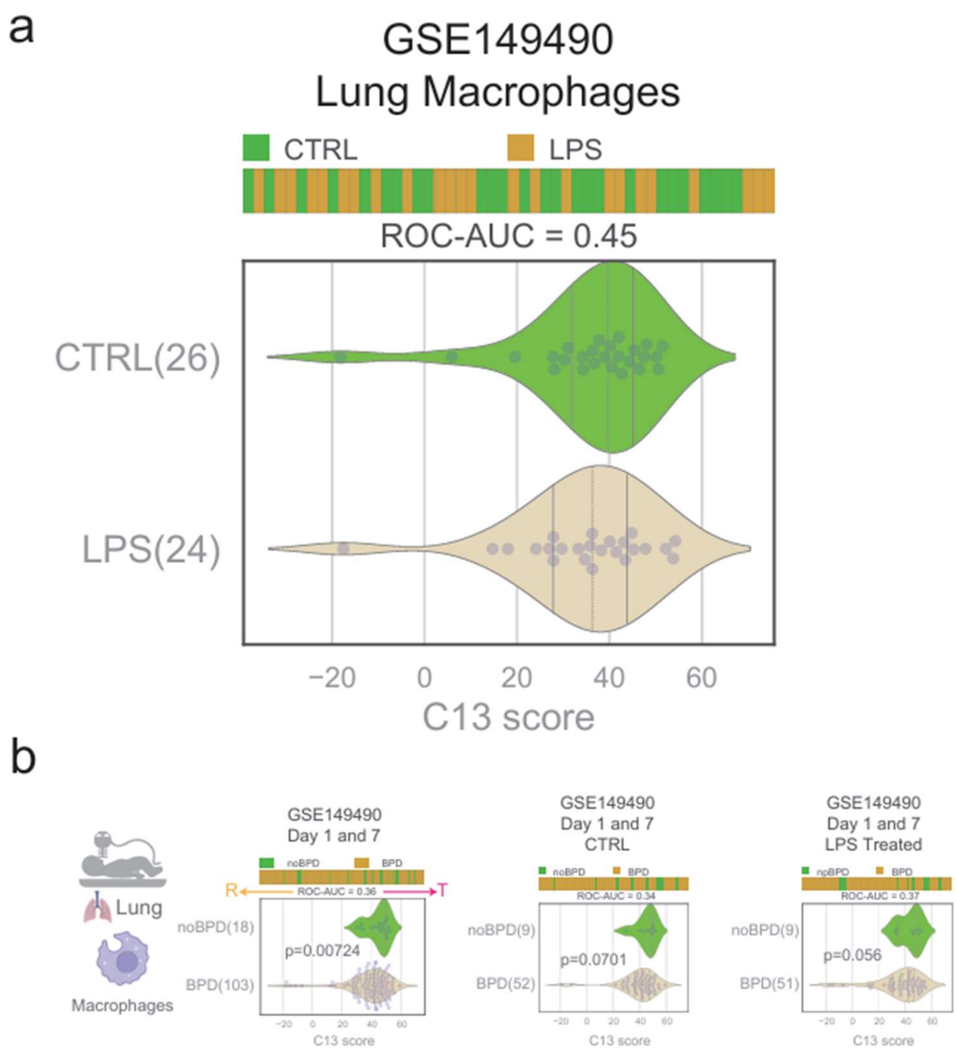

Supplemental Figure S2: SMaRT analysis of BAL-derived Lung Macrophages and BPD (GSE149490). Horizontal stacked bar plots (top) and violin (bottom) plots display the sub-classification of sample phenotypes. The x-axis shows the composite score based on clusters of genes (see Methods). Welch's two sample unpaired two-sided t-test is performed on the composite gene signature score to compute the p-values. Macrophage polarization is predicted using C13 score. (a) When combined, control and LPS-treated macrophages had similar reactivity/tolerance profiles. (b) When combined, control and LPS-treated macrophages exhibited increased reactivity with BPD (p=0.00724). When analyzed separately, there were non-significant trends towards reactive state with BPD in control (p=0.0701) and LPS-treated (p=0.056) BAL-derived lung macrophages.

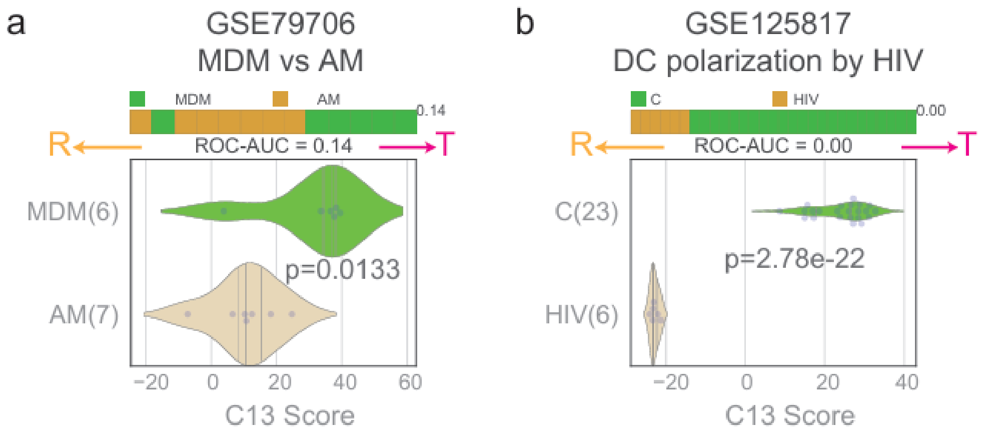

Supplemental Figure S3: Macrophage and Dendritic Cell polarization: (a) Alveolar macrophages (AM) are significantly more reactive compared to LPS polarized monocyte derived macrophage (MDM). (b) Dendritic cells are polarized by HIV to reactive state.
